# Transmission Dynamics of Hyper-Endemic Multi-Drug Resistant *Klebsiella pneumoniae* in a Southeast Asian Neonatal Unit: A Longitudinal Study With Whole Genome Sequencing

**DOI:** 10.3389/fmicb.2018.01197

**Published:** 2018-06-05

**Authors:** Pieter W. Smit, Nicole Stoesser, Sreymom Pol, Esther van Kleef, Mathupanee Oonsivilai, Pisey Tan, Leakhena Neou, Claudia Turner, Paul Turner, Ben S. Cooper

**Affiliations:** ^1^Centre for Tropical Medicine and Global Health, Nuffield Department of Medicine, University of Oxford, Headington, United Kingdom; ^2^Mahidol Oxford Tropical Medicine Research Unit, Faculty of Tropical Medicine, Mahidol University, Bangkok, Thailand; ^3^Nuffield Department of Clinical Medicine, University of Oxford, John Radcliffe Hospital (Level 5), Headington, United Kingdom; ^4^John Radcliffe Hospital Microbiology Laboratory, John Radcliffe Hospital (Level 7), Headington, United Kingdom; ^5^Cambodia-Oxford Medical Research Unit, Angkor Hospital for Children, Siem Reap, Cambodia

**Keywords:** *Klebsiella pneumoniae*, multidrug-resistance, colonization, whole genome sequencing, neonatal unit

## Abstract

**Background:** *Klebsiella pneumoniae* is an important and increasing cause of life-threatening disease in hospitalized neonates. Third generation cephalosporin resistance (3GC-R) is frequently a marker of multi-drug resistance, and can complicate management of infections. 3GC-R *K. pneumoniae* is hyper-endemic in many developing country settings, but its epidemiology is poorly understood and prospective studies of endemic transmission are lacking. We aimed to determine the transmission dynamics of 3GC-R *K. pneumoniae* in a newly opened neonatal unit (NU) in Cambodia and to address the following questions: what is the diversity of 3GC-R *K. pneumoniae* both within- and between-host; to what extent is high carriage prevalence driven by ward-based transmission; and to what extent can environmental contamination explain patterns of patient acquisition.

**Methods:** We performed a prospective longitudinal study between September and November 2013. Rectal swabs from consented patients were collected upon NU admission and every 3 days thereafter. Morphologically different colonies from swabs growing cefpodoxime-resistant *K. pneumoniae* were selected for whole-genome sequencing (WGS).

**Results:** One hundred and fifty-eight samples from 37 patients and 7 environmental sites were collected. 32/37 (86%) patients screened positive for 3GC-R *K. pneumoniae* and 93 colonies from 119 swabs were successfully sequenced. Isolates were resistant to a median of six (range 3–9) antimicrobials. WGS revealed high diversity; pairwise distances between isolates from the same patient were either 0–1 SNV or >1,000 SNVs; 19/32 colonized patients harbored *K. pneumoniae* colonies differing by >1000 SNVs. Diverse lineages accounted for 18 probable importations to the NU and nine probable transmission clusters involving 19/37 (51%) of screened patients. Median cluster size was five patients (range 3–9). Seven out of 46 environmental swabs (15%) were positive for 3GC-R *K. pneumoniae.* Environmental sources were plausible sources for acquisitions in 2/9 transmission clusters, though in both cases other patients were also plausible sources.

**Conclusion:** The epidemiology of 3GC-R *K. pneumoniae* was characterized by multiple introductions, high within- and between host diversity and a dense network of cross-infection, with half of screened neonates part of a transmission cluster. We found no evidence to suggest that environmental contamination was playing a dominant role in transmission.

## Introduction

Multi-drug resistant (MDR) *Klebsiella pneumoniae* is an increasing problem worldwide and is associated with poor patient outcomes, particularly in vulnerable groups such as neonates (Falade and Ayede, 2011; Snitkin et al., 2012; Munoz-Price et al., 2013; Hendrik et al., 2015; Naas et al., 2016; Stapleton et al., 2016; Turner et al., 2016). Whole genome sequencing (WGS) of clinical isolates has demonstrated the importance of the clonal spread in of *K.* *pneumoniae* in outbreak settings (Snitkin et al., 2012; Stoesser et al., 2014), and colonization is known to substantially increase the risk of infection (Martin et al., 2016; Gorrie et al., 2017). The underlying carriage dynamics of MDR *K.* *pneumoniae* in normal circumstances in high risk populations, however, remain poorly defined and longitudinal carriage studies in neonatal intensive care units and high endemicity settings are lacking. Importantly, it is not clear from previous reports whether reported transmission links between patients identified by WGS in outbreak settings represent unusual occurrences related to sporadic lapses in infection control or, on the contrary, whether frequent but hidden patient-to-patient transmission is the norm.

In a prospective 12-month study in a newly opened neonatal unit (NU) in Cambodia, we described hyper-endemic colonization with highly drug-resistant *K. pneumoniae* (Turner et al., 2016). Three-quarters of neonates in the unit were colonized with MDR *K. pneumoniae/oxytoca* and 2% (6/333) of these neonates developed hospital-acquired bacteraemia with a *K. pneumoniae* strain sharing the same antimicrobial susceptibility pattern as the colonizing strain. Here we focus on the first 2 months of data from this study and use WGS to provide a high-resolution snapshot of the underlying transmission dynamics. Such genomic approaches have been used for investigating transmission dynamics in an adult ICU in a high income setting (Gorrie et al., 2017), but have not been used to systematically investigate asymptomatic transmission in neonatal settings or in populations with a high level of endemic MDR *K. pneumoniae* infection. Longitudinal carriage studies are also lacking from developing country settings.

## Materials and Methods

The study took place in 2013 in a newly opened 11-bedded NU at the Angkor Hospital for Children, a non-governmental pediatric hospital in Siem Reap, Cambodia. The NU has two multi-patient rooms (one for intensive care and one for lower acuity patients; nurse:patient ratios over the study period were 0.63 and 0.40, respectively). There is a single isolation room. Angkor Hospital for Children has an active infection control program, including bleach-based environmental cleaning and monitoring of staff hand-hygiene compliance (Stoesser et al., 2013). Alcohol handrub is available by each bed and at ward entrances. Parents or guardians of neonates admitted to the NU during the study period were asked to provide consent. Rectal swabs were obtained from neonates entered into the study within 24 h of admission and twice weekly until NU discharge. Seven environmental sites within the NU were sampled twice weekly (six sink plug holes which were sampled by swabbing around plug hole and the unit computer keyboard). Here we consider only *K. pneumoniae* isolates resistant to cefpodoxime (a marker for multidrug-resistance) derived from swabs taken over a 7 weeks period following the opening of the NU. Patient swab results were not fed back to the NU clinicians.

Samples were processed and the extended spectrum beta-lactamase (ESBL)-producing phenotype defined as previously described (Turner et al., 2016). In brief, swabs were inoculated onto MacConkey agar (Oxoid). Morphologically distinct cefpodoxime and/or imipenem resistant isolates, as observed by the laboratory technicians, were sub-cultured; species identification as *K. pneumoniae* was undertaken using a panel of biochemical tests. All isolates were tested for susceptibility to the following antimicrobials by the disk diffusion method: ampicillin, co-amoxiclav, ceftriaxone, ciprofloxacin, gentamicin, co-trimoxazole, ceftazidime, chloramphenicol, imipenem, cefpodoxime, and nitrofurantoin. ESBL production was determined using the double-disk method (cefotaxime +/- clavulanate and ceftazidime +/- clavulanate), following Clinical & Laboratory Standards Institute guidelines (CLSI, 2015). Strains were considered MDR if non-susceptible to ≥3 antimicrobial categories (Magiorakos et al., 2012). The first 96 morphologically different colonies from swabs growing cefpodoxime-resistant *K. pneumoniae* were selected for WGS. DNA was extracted from pure *K. pneumoniae* sub-cultures using the FujiFilm QuickGene system (Fujifilm, Tokyo, Japan) as per the manufacturer’s instructions, with an additional mechanical lysis step (FastPrep-24; MP Biomedicals, Santa Ana, CA, United States) following chemical lysis. DNA extracts were sequenced on the HiSeq 2500 platform, generating 150 bp paired-end reads. Sequence data have been deposited in NCBI database (BioProject number: PRJNA395864).

To identify single nucleotide variants (SNVs), reads were mapped to the *K. pneumoniae* MGH78578 reference (GenBank: CP000647.1), and variants called as described previously (Stoesser et al., 2015b). *De novo* assemblies were generated using Velvet/Velvet optimiser (Zerbino and Birney, 2008), with *in silico* multilocus sequence typing (MLST) performed using BLASTn to query the assemblies with reference alleles/sequence types (STs) registered in the Pasteur *K. pneumoniae* MLST database^1^ (100% match over 100% length of reference sequence required to call an allele).

Phylogenetic trees were constructed using hierarchical clustering (Müllner, 2013) and ClonalFrameML (Didelot and Wilson, 2015). Plasmid replicons were detected using PlasmidFinder (Carattoli et al., 2014) (accessed May 10, 2016), using the SRST2 tool (Inouye et al., 2014). The ARG-ANNOT tool (accessed May 23, 2017) was used to detect known antibiotic-resistance genes (Gupta et al., 2014). Strains were defined as sequences with 0–1 SNV differences. Strain clusters were groups of different strains defined using an iterative procedure that placed two strains in the same cluster if they differed by ≤10 SNVs (Tong, 2015). Patient clusters were defined as patients harboring isolates of the same strain/strain cluster. Probable importation events were defined as a positive *K. pneumoniae* swab taken ≤48 h after NU admission. Probable acquisition events were defined as a first positive swab taken >48 h after NU admission together with initial negative swab. Plausible patient sources for acquisition events were considered to be neonates harboring the same strain/strain cluster who were on the NU prior to the acquisition event and discharged no more than 7 days prior to the acquiring patient’s admission date.

We used a permutation test to evaluate whether strains belonging to two STs had an increased likelihood of carrying the same plasmid or sharing phenotypic resistance to the same antibiotic if located within the same host (see Supplementary Material). We tested for associations between time since unit admission and the number of plasmid replicons, resistance genes, and phenotypic resistances within a sample using random effects Poisson regression models (adjusting for within-host clustering effects). Statistical analysis was performed in R (Tong, 2015).

The study was approved by the Angkor Hospital for Children Institutional Review Board and the Oxford Tropical Ethics Committee.

## Results

**FIGURE 1:**
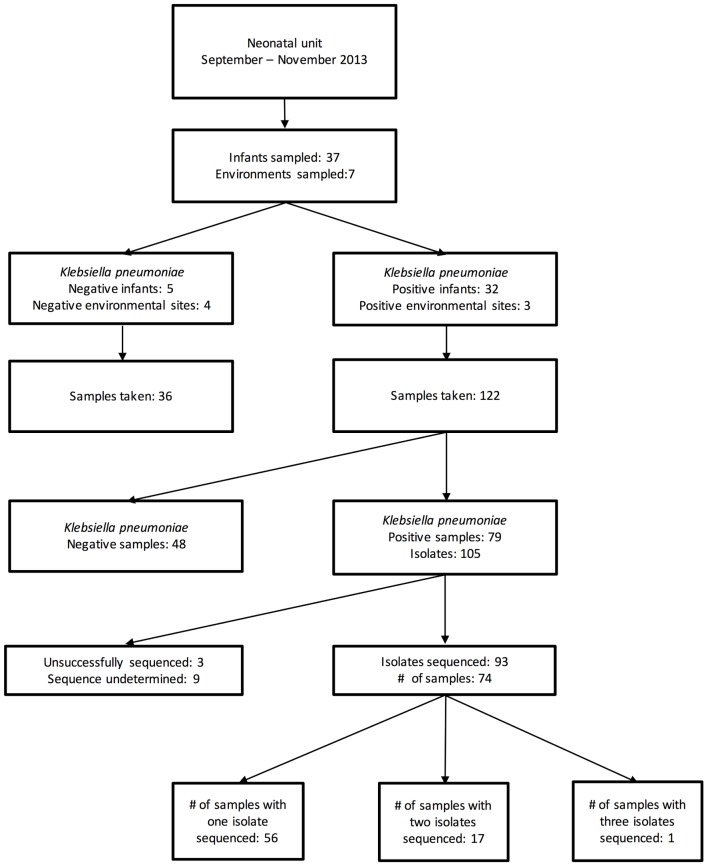
Flow diagram of study sampling strategy.

During the 7 weeks period, 158 samples from 37 patients and 7 environmental locations were collected. Of these, 79 samples [from 32 patients and 3 environmental sites (all sinks)] were cefpodoxime-resistant *K. pneumoniae* culture-positive, generating 105 MDR *K. pneumoniae* isolates (**Figure 1**). The first 96 of these isolates were selected for sequencing.

All 32 colonized patients were born outside the NU, with 16 born at a hospital, 13 at a health center and three at home. None of these patients developed culture-proven *K. pneumoniae* invasive infection during the study timeframe.

Seventeen neonates (46% of those screened, 53% of those who were ever carrying MDR *K. pneumoniae*) were found to be colonized within 24 h of NU admission, eight had their first positive sample at 1–3 days after admission and seven had a first positive sample 3–14 days after NU admission (median: 4 days). Of the seven environmental sites that were screened, three sink basins located in the isolation room, dirty utility, and milk kitchen room were intermittently positive. The first sink became positive 12 days after the first patient arrived.

### Antimicrobial Resistance

**FIGURE 2:**
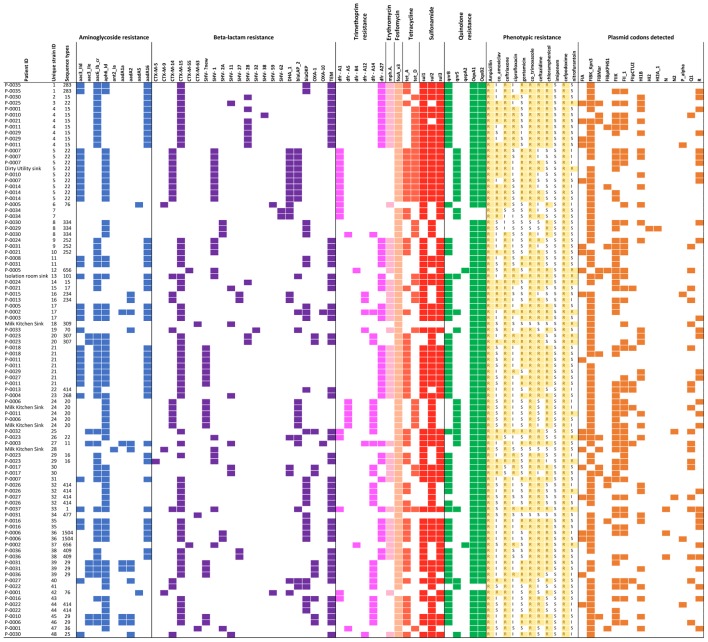
Overview of all isolates with colored blocks representing the presence of antimicrobial resistance genes, phenotypic resistance, or plasmid replicons. The list is sorted per unique strain ID (cut-off used: 0–1 single nucleotide variant difference), highlighting variations in phenotypic resistance and plasmid replicons detected among genetically identical strains. Isolates with missing sequence type did not provide a 100% match over 100% length of reference sequence to generate a valid ST.

Of 93 successfully sequenced isolates, all were MDR, with phenotypic resistance to a median of six of the 11 antibiotics tested (**Figure 2**); 17 isolates were resistant to ≥8 antibiotics and 22 were resistant to all antibiotic classes tested except nitrofurantoin and quinolones. Only one isolate did not have an ESBL phenotype. No isolates were resistant to imipenem. Amongst the 18 samples from which multiple colonies were selected and successfully sequenced, all morphologically distinguishable colonies had a different antimicrobial resistance profile.

A total of 21 resistance genes (49 allelic variants) were detected, and isolates harbored an average of 14 allelic variants of resistance genes (range: 5–24). The relationship between phenotypic resistance and the presence of known resistance genes is shown in **Supplementary Figures 1, 2**. A histogram showing the frequency of different phenotypic antimicrobial resistance combinations is shown in **Supplementary Figure 3**.

### Whole Genome Sequencing

Sixty-one strains were identified amongst the 93 successfully sequenced colonies. Thirteen of these were represented by ≥1 isolate, and 9 strains were found in two or more patients. WGS revealed high genetic diversity among the sequenced isolates (median 13,580 SNVs, range 0–24,052). Twenty-five different STs were identified from 69 isolates where ST could be established; 10 of these STs were identified only once.

### Within- and Between-Host Diversity

**FIGURE 3:**
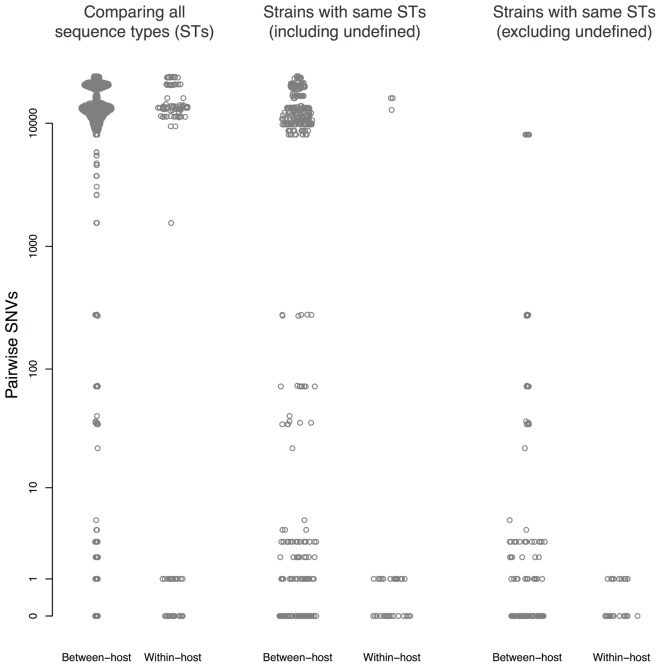
Pairwise single nucleotide variants (SNVs) between sequences. Results are shown for all pairwise comparisons **(Left)**, including pairs with different STs **(center)**, and excluding pairs with different STs or undefined ST **(right)**. Points are jittered in proportion to the kernel density estimate.

Thirty patients (94%) and three environmental sites had more than one isolate sequenced. Amongst these, within-host pairwise distances were either 0–1 or >1,000 SNVs (**Figure 3**). There were 45 sequences that differed ≤1 SNV from other sequenced isolates from the same patient, and 55 sequences that differed by ≤10 SNVs from sequences from a different patient.

**FIGURE 4:**
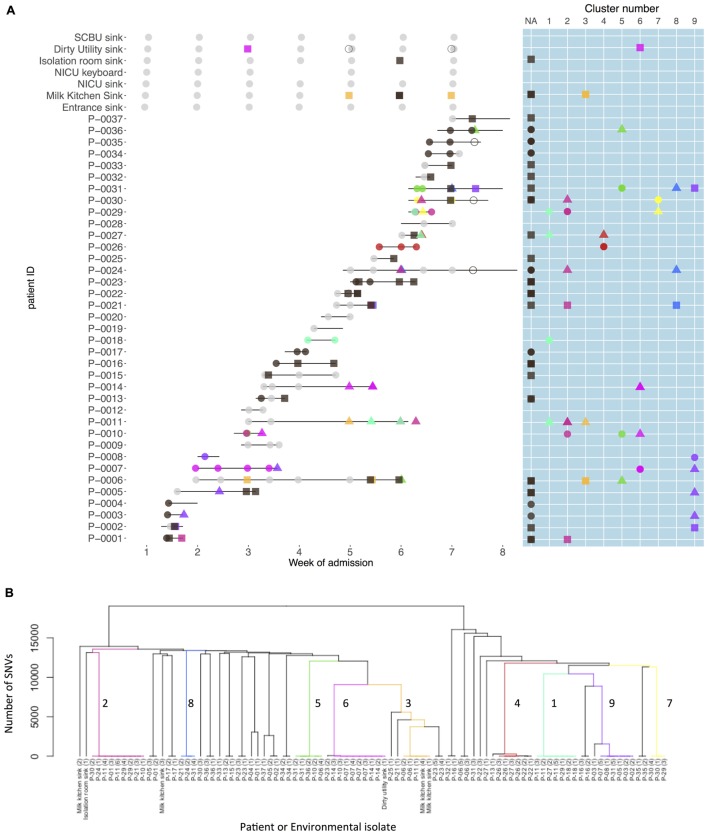
Patient carriage and environmental contamination with clustered and unclustered multidrug-resistant (MDR) *K. pneumoniae* strains and associated phylogenetic tree. **(A)** Shows the timeline for individual neonatal admissions (lines) and the identification of clustered isolates. Light gray dots represent negative samples, dark gray indicates samples positive for MDR *K. pneumoniae* but with non-clustered strains. Other colors depict genetically defined strain clusters. Shapes correspond to inferred importation/acquisition events; overlapping shapes indicate that different strains were detected from the same patient or environmental sample. Where the same colored shape is represented consecutively, this represents persistent carriage with a strain that was either imported (consecutive circles) or acquired on the neonatal unit (consecutive squares or triangles). **(B)** Hierarchical phylogenetic tree of the isolates included in this study. Colors depict isolates from the same genetically defined strain clusters as shown in **A**.

Nine strain clusters were detected (cluster defined by ≤10 SNVs, see “Materials and Methods” section for detailed description), and identified amongst 19/32 colonized patients in patient clusters ranging in size from three to nine patients (median 5). Ten patients (31%) were part of multiple patient clusters (**Figure 4**). In a sensitivity analysis where we varied the SNV threshold in the strain cluster definition from 10 to either 5 or 20 we identified identical strain and patient clusters.

### Strain Importations and Acquisitions

Among the 32 *K. pneumoniae* positive patients, 18 (56%) represented probable importations. However, 11 of these patients (61%) also showed evidence of acquiring additional *K. pneumoniae* strains during their NU admission. There were 52 such possible acquisitions (defined by a first positive swab for a strain at least 48 h after unit admission preceded by at least one negative swab for that strain/strain cluster) which occurred in 25 patients. In 22 of these 52 possible acquisitions a plausible source was identified. Environmental sources were identified as plausible sources for acquisitions in 2/9 clusters, though in both cases other patients were also plausible sources.

### Plasmids

We identified 15 known plasmid replicon sequences. All isolates were positive for at least one plasmid replicon, with an average of 3.4 replicon types per isolate (range 1–6) (**Figure 2** and **Supplementary Table 1**). Identical strains which carried the same resistance genes, had different plasmids identified. Neither of the plasmid-mediated colistin-resistance genes, *mcr-1* and *mcr-2*, were detected.

We found no evidence of systematic changes in the number of distinct plasmids, the number of resistance genes, or the number of antibiotics to which isolates were phenotypically resistant over the course of a patient’s stay (**Supplementary Figure 4** and **Supplementary Table 2**). We found no evidence of an association between the number of distinct plasmid replicons detected and either the number of resistance genes identified or the number of antibiotics to which isolates were phenotypically resistant, though (as expected) there was a positive correlation between the number of resistance genes and the phenotypic resistance count (**Supplementary Figure 5**). We found no evidence that strains from two STs were more likely to harbor the same plasmid if located within the same patient (**Supplementary Table 3**). Similarly, with the exception of nitrofurantoin, there was no statistical evidence of within-host transfer of phenotypic resistance between different STs (**Supplementary Table 4**).

## Discussion

The high frequency with which MDR *K. pneumoniae* isolates differing by ≤10 SNVs were recovered from different neonates together with the timing of probable acquisition events suggested frequent cross-infection between neonates and a high background rate of importation of new strains [almost half of the neonates were colonized on admission; 32/37 (86%) were carriers at some point]. These apparently high transmission rates occurred despite relatively good levels of infection control and antibiotic stewardship (Stoesser et al., 2013). Individuals were commonly involved in multiple strain clusters and the high prevalence of co-infection with genetically distant lineages of MDR *K. pneumoniae* points to a complex epidemiology that differs substantially from both that found in prospective WGS carriage studies of *K. pneumoniae* in a high-income adult intensive care unit setting (Gorrie et al., 2017), and that suggested by older carriage studies in high income neonatal intensive care units (Goldmann et al., 1978; Toltzis et al., 2001). Because the decision to perform this study was not motivated by an unusual clusters of cases, the results strongly suggest that such high rates of transmission of asymptomatic infection may be typical in NUs where MDR *K. pneumoniae* is endemic.

High risk clones that are associated with extensive outbreaks, such as ST15 (Zhou et al., 2016), was associated with relatively low level within-ward transmission in this study (9 isolates). Dominant strains in other parts of the world such as ST11 (*n* = 1) (Yan et al., 2015), ST258 (*n* = 0) (Deleo et al., 2014), ST147 (*n* = 0) (Pan et al., 2015) were practically not observed in this study, suggesting a wide variety of other prevalent MDR *K. pneumoniae* endemic STs in Cambodia.

Quantifying the role of environmental contamination in transmission within hospital units is difficult, as presence of contamination does not necessarily imply such contamination is important for transmission. Randomized trials (that reduce such contamination) are needed to definitively establish a causal link and quantify the effects on transmission. However, contaminated sinks have been associated with previous outbreaks and it has been suggested that more intensive cleaning protocols might be needed (Lowe et al., 2012). In our study, environmental sites showed only intermittent contamination, although three out of six sinks screened positive on at least one occasion. However, none of these sites showed persistent colonization with a single lineage and rates for positivity were low suggesting that cleaning protocols were effective and that the environment may have played a limited role in the transmission dynamics.

Strengths of this work include the use of systematic and high frequency screening of most neonates in the unit, highly discriminatory typing, and the sequencing of multiple morphologically distinct colonies grown from the same swabs. Without these three approaches we would have had a far more limited view of the complex transmission network.

A limitation was the inability to associate specific resistance genes with specific plasmids. This cannot usually be done reliably without fully resolving plasmid structures using long-read sequencing (Lowe et al., 2012). Also, by limiting our analysis to cefpodoxime-resistant *K. pneumoniae* we have only a partial epidemiological picture. It is possible, for example, that some of the apparent acquisition events of MDR *K. pneumoniae* where no plausible source was identified may have resulted from the transfer of mobile genetic elements between patients conferring cefpodoxime-resistance to previously cefpodoxime-sensitive strains, as a result of transfer from other colonizing species which were not surveyed, or as a result of failure to completely characterize within-host diversity. In relation to the latter, morphology may not be the most sensitive way to distinguish within-host diversity; multiple picks, irrespective of colonial morphology, may uncover significant additional diversity (Stoesser et al., 2015a).

There are three key findings from this study that have important implications for attempts to control the spread of MDR *K. pneumoniae*. The first is to suggest that super-infection with diverse lineages of *K. pneumoniae* might be the norm rather than exception in neonatal populations, highlighting the importance of considering the carrier state and taking more than one sample per patient, and investigating more than one colony within a sample culture, when trying to understand transmission chains of *K. pneumoniae* (similar considerations have been shown to be important for ESBL-producing *Escherichia coli* (Stoesser et al., 2015a). The second is to highlight the high degree of cross-infection in the NU despite good infection control and antibiotic stewardship practices. The third is to show that high numbers of infants are likely to have be already colonized when admitted to the unit, suggesting acquisition in the community may be important in this population. Together these observations highlight the scale of the challenge in halting the spread of MDR *K. pneumoniae* in endemic settings; our findings emphasize the importance and difficulty of both finding effective interventions to impede its nosocomial spread as well as measures to reduce the chance that infants are carrying this organism when admitted to hospital. Currently we are lacking strategies of proven effectiveness to do either.

## Ethics Statement

The study was carried out in accordance with the recommendations of Good Clinical Practice (ICH). The protocol was approved by the Angkor Hospital for Children Institutional Review Board and the Oxford Tropical Ethics Committee. Parents of all subjects gave written informed consent in accordance with the Declaration of Helsinki.

## Author Contributions

BC, PaT, and CT initiated and oversaw the collection, data analysis and writing up of the study. LN, SP, and MO collected the samples, performed laboratory tests and were involved in data analysis and writing up. EvK, PiT, PS, and NS were responsible for the analysis of the data and writing up of the study.

## Conflict of Interest Statement

The authors declare that the research was conducted in the absence of any commercial or financial relationships that could be construed as a potential conflict of interest.

## Footnotes

1 http://bigsdb.pasteur.fr/klebsiella/klebsiella.html
