# Supplementary material for: Transmission Dynamics of Hyper-Endemic Multi-Drug Resistant Klebsiella pneumoniae in a Southeast Asian Neonatal Unit: A Longitudinal Study With Whole Genome Sequencing
Source: Front Microbiol. 2018 Jun 5;9:1197. doi: 10.3389/fmicb.2018.01197 (PMC5996243; doi:10.3389/fmicb.2018.01197)

Supplementary Figure 1. Heatmap showing the percentage of isolates showing phenotypic resistance to a given antibiotic which carry each resistance gene.

Resistance genes are grouped by antimicrobial class on the x-axis, with resistance phenotypes grouped by antimicrobial class on the y-axis. Sample size for each phenotypic or genotypic marker is shown (n=) on both axes. The areas where a direct relationship between resistance genotype and phenotype might be expected are outlined in black.

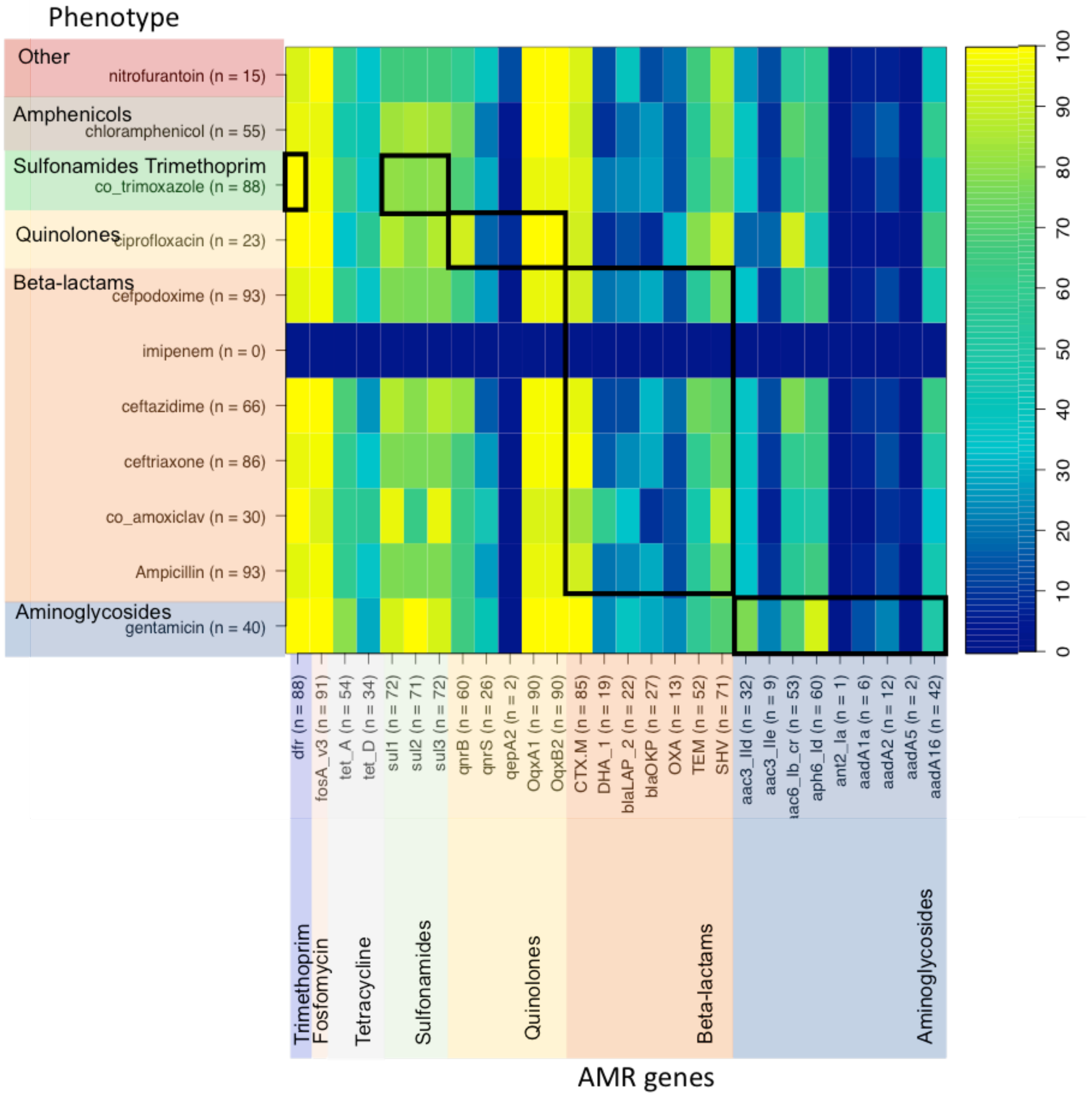

Supplement: Supplementary file 1 [file Image_1.PDF]
