# Supplementary material for: Transmission Dynamics of Hyper-Endemic Multi-Drug Resistant Klebsiella pneumoniae in a Southeast Asian Neonatal Unit: A Longitudinal Study With Whole Genome Sequencing
Source: Front Microbiol. 2018 Jun 5;9:1197. doi: 10.3389/fmicb.2018.01197 (PMC5996243; doi:10.3389/fmicb.2018.01197)

Supplementary Figure 3. Frequency plot of isolates for classes of phenotypic antimicrobial resistance combinations.

Each bar represents the % of isolates with one phenotypic resistance combination. The broad antibiotic classes to which isolates are resistant are identified by the colors crossing the vertical bars (note that all isolates are resistant to the beta-lactam Cefpodoxime in accordance with the selection criteria).

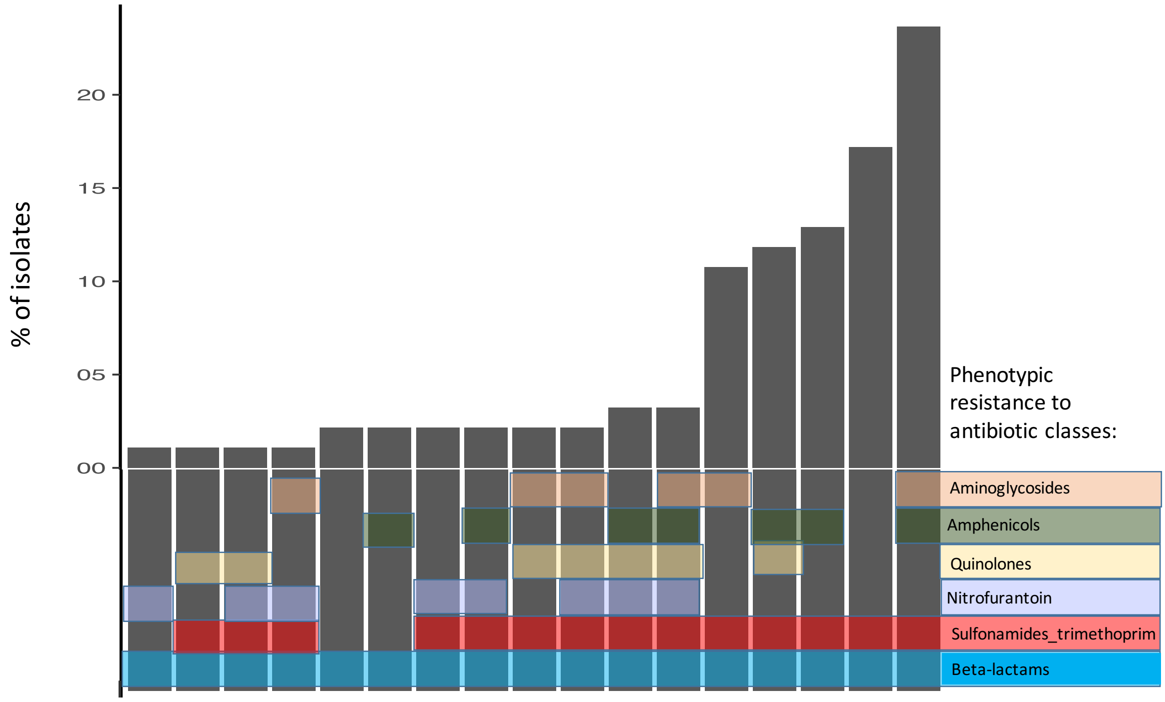

Supplement: Supplementary file 3 [file Image_3.PDF]
