# Supplementary material for: Transmission Dynamics of Hyper-Endemic Multi-Drug Resistant Klebsiella pneumoniae in a Southeast Asian Neonatal Unit: A Longitudinal Study With Whole Genome Sequencing
Source: Front Microbiol. 2018 Jun 5;9:1197. doi: 10.3389/fmicb.2018.01197 (PMC5996243; doi:10.3389/fmicb.2018.01197)

Supplementary Figure 4. The number of plasmid replicons, the resistance gene count, and the phenotypic resistance count detected per isolate by days since admission to the neonatal unit when the isolate was taken.
Points in red correspond to infants who were admitted to another hospital, or another department in the same hospital, prior to admission to the NICU. Points in blue correspond to infants for who were first admitted to the neonatal unit. Within each color, different symbols correspond to different patients. Jitter has been added to enable overlapping points to be seen.

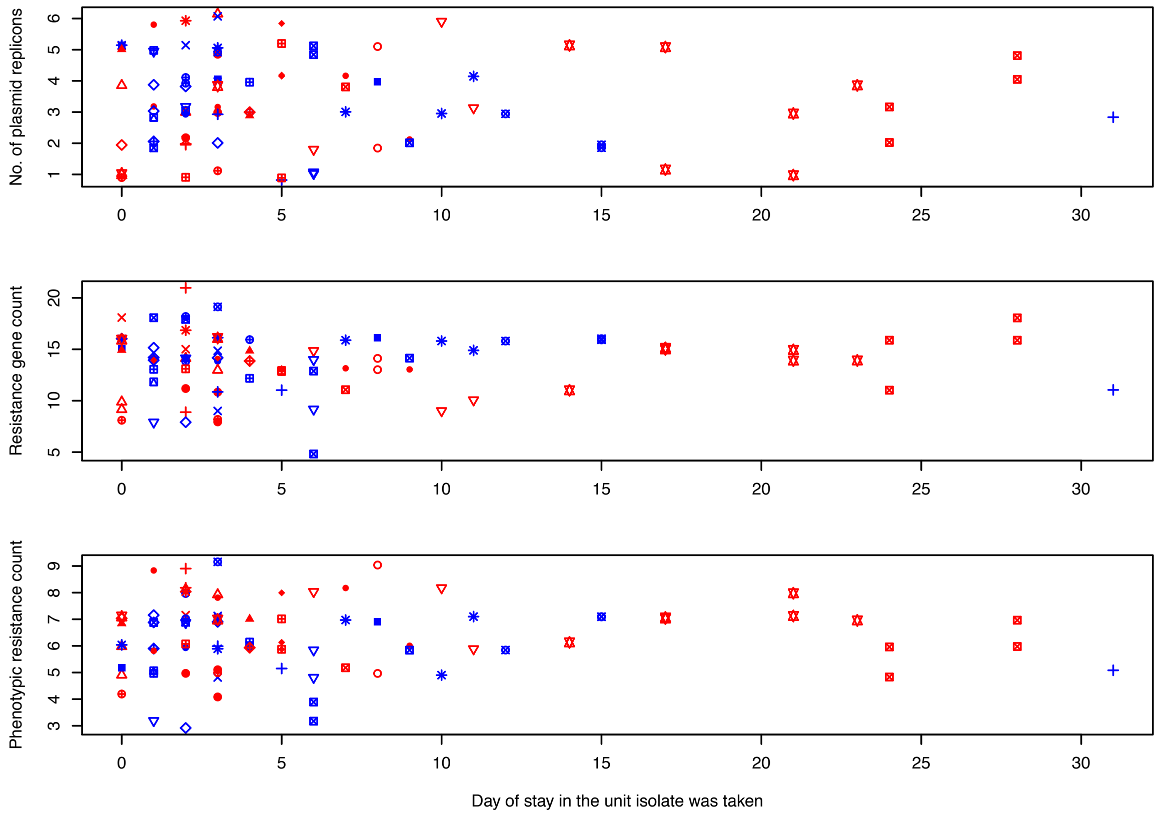

Supplement: Supplementary file 4 [file Image_4.PDF]
