# Supplementary material for: Transmission Dynamics of Hyper-Endemic Multi-Drug Resistant Klebsiella pneumoniae in a Southeast Asian Neonatal Unit: A Longitudinal Study With Whole Genome Sequencing
Source: Front Microbiol. 2018 Jun 5;9:1197. doi: 10.3389/fmicb.2018.01197 (PMC5996243; doi:10.3389/fmicb.2018.01197)

Supplementary Figure 5. The relationships between the number of plasmid replicons, the number of resistance genes, and the phenotypic resistance count. Each point corresponds to one sequenced strain (with added jitter). Colors and symbols have the same interpretation as in Supplementary Figure 4. There was no evidence of an association between the number of plasmid replicons and the number of resistance genes (Pearson's correlation coefficient 0.02, 95% CI [-0.19, 0.23]), or between plasmid replicons and the phenotypic resistance count (-0.03 [-0.24, 0.18]), but the phenotypic resistance count was correlated with the number of resistance genes (0.62 [0.47, 0.73]).

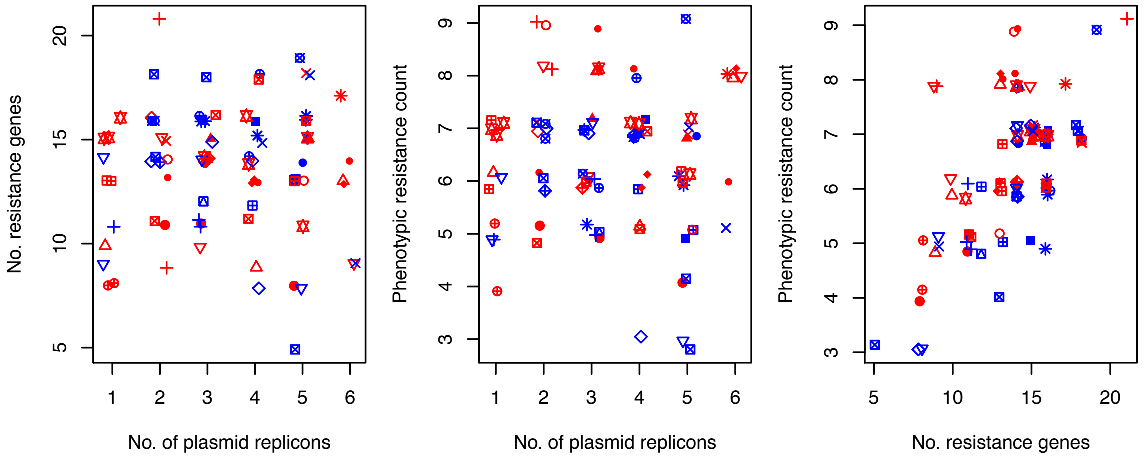

Supplement: Supplementary file 5 [file Image_5.PDF]
