# Supplementary material for: Transmission Dynamics of Hyper-Endemic Multi-Drug Resistant Klebsiella pneumoniae in a Southeast Asian Neonatal Unit: A Longitudinal Study With Whole Genome Sequencing
Source: Front Microbiol. 2018 Jun 5;9:1197. doi: 10.3389/fmicb.2018.01197 (PMC5996243; doi:10.3389/fmicb.2018.01197)
Supplement: Supplementary file 6 [file Table_1.PDF]

Supplementary Table 1. Plasmid replicons detected among all isolates included in the study.

This shows the frequency of the plasmid replicons that were detected (# isolates), genotypic and phenotypic antimicrobial resistance profiles in terms of the number of phenotypic resistance categories and the number of resistance genes (observed range in isolates where plasmid replicon was found), and the number of plasmid replicons detected among the isolates harbouring each plasmid replicon (range in isolates where plasmid replicon was found).

Plasmid	# isolates	# phenotypic resistance categories (range)	# resistance genes (range)	# plasmids (range)
FIBK_Kpn3	82	(3-8)	(9-17)	(1-6)
FIIK	57	(3-9)	(5-21)	(1-6)
FII_1	45	(3-9)	(9-18)	(3-6)
R	30	(4-8)	(9-16)	(3-6)
HI1B	29	(3-9)	(5-21)	(2-6)
Q1	20	(3-9)	(5-19)	(2-6)
FIA	19	(5-7)	(11-18)	(4-5)
FIBMar	11	(3-9)	(5-18)	(3-6)
FIBpKPHS1	8	(3-3)	(8-8)	(4-4)
FIIpCTU2	7	(3-3)	(8-8)	(4-4)
P_alpha	4	(6-9)	(13-19)	(5-5)
N	3	(5-8)	(9-17)	(6-6)
N2	2	(5-8)	(11-16)	(3-3)
HI2	1	(3-9)	(5-19)	(3-6)
HI2A_1	1	(3-9)	(5-19)	(2-5)
