# Supplementary material for: Transmission Dynamics of Hyper-Endemic Multi-Drug Resistant Klebsiella pneumoniae in a Southeast Asian Neonatal Unit: A Longitudinal Study With Whole Genome Sequencing
Source: Front Microbiol. 2018 Jun 5;9:1197. doi: 10.3389/fmicb.2018.01197 (PMC5996243; doi:10.3389/fmicb.2018.01197)
Supplement: Supplementary file 7 [file Table_2.DOCX]

Supplementary Table 2. Results of mixed effects Poisson regression to evaluate factors associated with the number of plasmid replicons per isolate, the resistance gene count, and the phenotypic resistance count.

All models adjusted for within-host clustering through a random effects intercept term.

| Outcome | Covariate | Relative Risk | 95% CI | p-value |
| --- | --- | --- | --- | --- |
| Number of plasmid replicons | Days from NU admission to isolate | 1.00 | (0.98, 1.02) | 0.99 |
|  | Direct admission to NU | 1.00 | (0.76, 1.30) | 0.99 |
|  | Female | 1.04 | (0.81, 1.33) | 0.78 |
|  | Resident in Siem Reap | 0.89 | (0.70, 1.15) | 0.37 |
|  | Premature | 0.89 | (0.61, 1.29) | 0.55 |
|  |  |  |  |  |
| Resistance gene count | Days from NU admission to isolate | 1.00 | (0.99, 1.01) | 0.38 |
|  | Direct admission to NU | 1.04 | (0.91, 1.19) | 0.59 |
|  | Female | 0.88 | (0.77, 1.00) | 0.05 |
|  | Resident in Siem Reap | 0.92 | (0.82, 1.05) | 0.22 |
|  | Premature | 1.05 | (0.87, 1.26) | 0.63 |
|  |  |  |  |  |
| Phenotypic resistance count | Days from NU admission to isolate | 1.00 | (0.98, 1.01) | 0.81 |
|  | Direct admission to NU | 0.92 | (0.75, 1.12) | 0.39 |
|  | Female | 0.97 | (0.80, 1.16) | 0.73 |
|  | Resident in Siem Reap | 0.98 | (0.81, 1.18) | 0.80 |
|  | Premature | 1.06 | (0.81, 1.38) | 0.67 |
