# Supplementary material for: Transmission Dynamics of Hyper-Endemic Multi-Drug Resistant Klebsiella pneumoniae in a Southeast Asian Neonatal Unit: A Longitudinal Study With Whole Genome Sequencing
Source: Front Microbiol. 2018 Jun 5;9:1197. doi: 10.3389/fmicb.2018.01197 (PMC5996243; doi:10.3389/fmicb.2018.01197)
Supplement: Supplementary file 8 [file Table_3.DOCX]

## Permutation test

A permutation test was used to test the null hypothesis that strains belonging to two sequence types do not have an altered likelihood of carrying the same plasmid or sharing phenotypic resistance to the same antibiotic if located within the same patient. The code and full documentation for the permutation test can be found at the github repository https://github.com/m6thu/Plasmid_ST_Permutation_Test

## Supplementary Table 3. Plasmid permutation test results.

| **Plasmid** | **Incompatability Group** | **p-value** |
| --- | --- | --- |
| FIA | FIA | 0.82 |
| FIBK | FIB | 0.79 |
| FIBMar | FIB | 0.47 |
| FIBpKPHS1 | FIB | 0.91 |
| FIIK | FII | 0.58 |
| FII1 | FII | 0.44 |
| FIIpCTU | FII | 0.57 |
| HI1B | HI1 | 0.64 |
| HI2 | HI2 | 1.00 |
| HI2A | HI2 | 1.00 |
| N | N | 1.00 |
| N2 | N | 0.64 |
| P-alpha | P-alpha | 0.90 |
| Q1 | Q | 0.69 |
| R | R | 0.14 |
