# Supplementary material for: Transmission Dynamics of Hyper-Endemic Multi-Drug Resistant Klebsiella pneumoniae in a Southeast Asian Neonatal Unit: A Longitudinal Study With Whole Genome Sequencing
Source: Front Microbiol. 2018 Jun 5;9:1197. doi: 10.3389/fmicb.2018.01197 (PMC5996243; doi:10.3389/fmicb.2018.01197)
Supplement: Supplementary file 9 [file Table_4.DOCX]

## Supplementary Table 4. Antibiotic resistance phenotype permutation test results.

| **Antibiotic** | **Abbreviation** | **Antibiotic Group** | **p-value (tight)*** | **p-value (loose)** * |
| --- | --- | --- | --- | --- |
| Ampicillin | amp | Beta-lactam | 1.00 | 1.00 |
| Co-amoxiclav | amc | Beta-lactam | 0.62 | 0.60 |
| Ceftriaxone | cro | Cephalosporins | 0.49 | 0.69 |
| Ciprofloxacin | cip | Quinolones | 0.22 | 0.59 |
| Gentamicin | cn | Aminoglycosides | 0.10 | 0.17 |
| Co-trimoxazole | sxt | Sulfanomides | 1.00 | 1.00 |
| Ceftazidime | caz | Cephalosporins | 0.41 | 0.75 |
| Chloramphenicol | c | Chloramphenicol | 0.30 | 0.23 |
| Imipenem | ipm | Carbapenem | 1.00 | 1.00 |
| Cefpodoxime | cpd | Cephalosporins | 1.00 | 1.00 |
| Nitrofurantoin | f | nitrofuran | 0.10 | 0.01 |

*Tight defines intermediate resistance as sensitive, while loose considers intermediate as resistant.
